# New insights into copper homeostasis in filamentous fungi

**DOI:** 10.1007/s10123-019-00081-5

**Published:** 2019-05-15

**Authors:** Martzel Antsotegi-Uskola, Ane Markina-Iñarrairaegui, Unai Ugalde

**Affiliations:** Microbial Biochemistry Laboratory, Department of Applied Chemistry, Faculty of Chemistry, University of the Basque Country, San Sebastian, Spain

**Keywords:** Copper, Homeostasis, Ctr, P-type ATPase, Transcription factor

## Abstract

Copper is a metal ion that is required as a micronutrient for growth and proliferation. However, copper accumulation generates toxicity by multiple mechanisms, potentially leading to cell death. Due to its toxic nature at high concentrations, different chemical variants of copper have been extensively used as antifungal agents in agriculture and medicine. Most studies on copper homeostasis have been carried out in bacteria, yeast, and mammalian organisms. However, knowledge on filamentous fungi is less well documented. This review summarizes the knowledge gathered in the last few years about copper homeostasis in the filamentous fungi *Aspergillus fumigatu*s and *Aspergillus nidulans*: The mechanism of action of copper, the uptake and detoxification systems, their regulation at the transcriptional level, and the role of copper homeostasis in fungal pathogenicity are presented.

## Introduction

Virtually, all organisms depend on metal ions as catalysts, as structural elements in proteins, in electron transfer reactions, or as messengers. The first-row transition metals, cobalt (Co), copper (Cu), iron (Fe), manganese (Mn), and nickel (Ni), possess specific redox potential characteristics which are unavoidably required in key biological processes (Gerwien et al. 2018; Nevitt et al. 2012).

The availability of these metals must be maintained within a narrow range of concentrations, as an excess can easily result in metal toxicity (Blatzer and Latge 2017). All organisms have developed precise mechanisms to respond to fluctuations in extracellular metal availability enabling homeostatic regulation for each metal ion species. This is achieved by accurate sensing of extracellular and intracellular levels, and the activation of stimulus-response pathways that maintain intracellular levels within the safety range (Nevitt et al. 2012).

Copper functions as a cofactor in enzymes involved in processes such as cellular respiration (cytochrome-c oxidase), free radical detoxification (superoxide dismutase), pigmentation (tyrosinase), collagen maturation (lysyl oxidase), and iron acquisition (Ding et al. 2014; Lutsenko 2010; Nevitt et al. 2012). However, accumulation of copper beyond homeostatic capacity generates toxicity. An excess of free cytosolic copper ions leads to the inactivation of other metalloenzymes by metal displacement, the inappropriate engagement with intracellular metalophilic ligands such as Fe-S clusters, and the generation of reactive oxygen species (ROS) through Fenton chemistry (Fridovich 1983; Macomber and Imlay 2009). The essential, yet toxic, nature of copper demands for sensitive control mechanisms over intracellular Cu levels.

Most studies in copper homeostasis have been carried out on mammalian, bacteria, and yeast model systems. The model yeast *Saccharomyces cerevisiae* has served as an important reference for other lower eukaryotes (Balamurugan and Schaffner 2006), including pathogens like *Cryptococcus neoformans* and *Candida albicans* (Ballou and Wilson 2016). In these models, copper is also a recognized virulence factor (Zhang et al. 2016). A number of recent publications have described copper import and export mechanisms in *Aspergillus fumigatus*, a common airborne fungal pathogen (Blatzer and Latge 2017), responsible of severe invasive aspergillosis in immunocompromised patients (Cai et al. 2017).

In this review, we present an overview of recently published findings on different aspects of copper homeostasis in filamentous fungi, which constitute a major group of microorganisms associated with agricultural, food, clinical, and environmental issues. We will pay special attention to *Aspergillus fumigatus*, the model organism *Aspegillus nidulans*, and the plant pathogen *Botrytis cinerea*.

## Copper import across membranes

Fungal hyphae must acquire copper from the environment and maintain intracellular concentration within the micromolar range. Copper is generally internalized through low- and high-affinity uptake systems, depending on extracellular copper concentration. The most studied copper transport system is that of *S*. *cerevisiae*, and studies in other fungi have been shown to be considerably similar (Balamurugan and Schaffner 2006).

The membrane-associated copper transporting protein (Ctr) family is omnipresent in all eukaryotes and they all share high specificity for copper. Ctr proteins are relatively small (18–30 kDa) and can contain up to three transmembrane domains (Petris 2004; Puig et al. 2002). Copper binding or acquisition motifs (Mets) are located in the extracellular N-terminal region and are rich in methionine (MxxM or MxM) (Jiang et al. 2005). Another methionine-rich motif MxxxM, located in a transmembrane domain, has been proved to be essential for copper ion (Cu^+^) transport through the membrane (Puig et al. 2002). Ctr monomers assemble forming a trimer, as Cu^+^ transport requires Ctr protein multimerization to create a pore that drives the copper ion through the membrane (De Feo et al. 2009). Cu^+^ enters the cell by passive transport, facilitated by an extremely low intracellular copper concentration (Balamurugan and Schaffner 2006). Copper is normally present in the environment in two oxidation forms: Cu^2+^ and Cu^+^. However, only Cu^+^ is recognized as a substrate by the transporters. The extracellular Cu^2+^ is reduced by plasma membrane reductases prior to internalization. These plasma membrane reductases are equally widespread in eukaryotes (Garcia-Santamarina et al. 2018; Marvin et al. 2004).

**Fig. 1:**
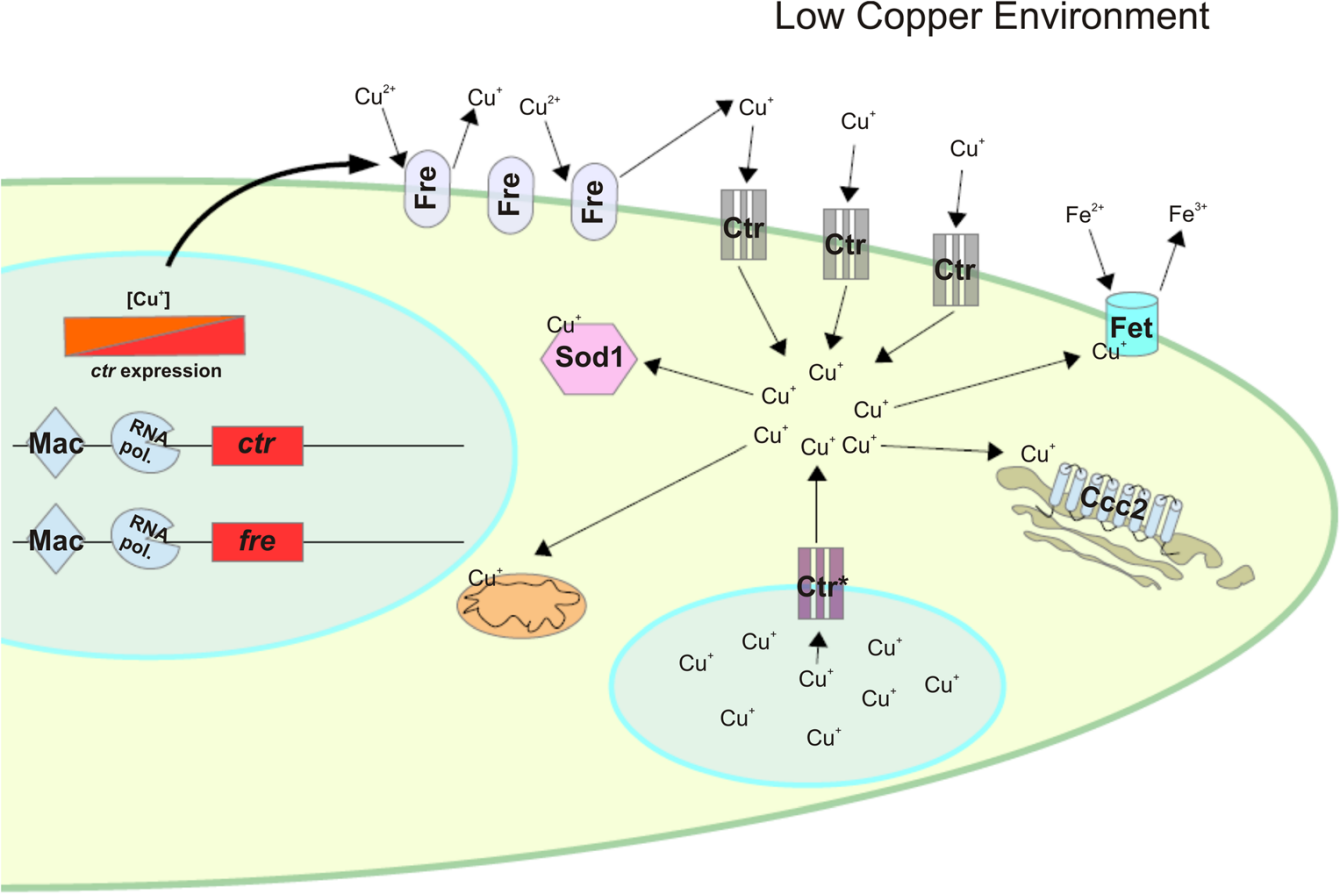
Copper homeostasis scheme for *A*. *nidulans* and *A*. *fumigatus*, under conditions of low copper availability. TF MacA binds to DNA enabling the transcription of the copper transporting proteins (*ctr*) and the plasma membrane copper reductase (*fre*) coding genes. The plasma membrane copper reductases (Fre) reduce environmental copper from Cu^2+^ to Cu^+^. Subsequently, the copper transporting proteins (Ctr) introduce copper to the cell. Within the cell, Cu^+^ ions are distributed to the mitochondria as cofactor of the electron transport chain enzymes, the superoxide dismutase Sod1 or the ferroxidase Fet, essential for Reductive Iron Assimilation (RIA). Copper is also distributed to the P-type ATPase Ccc2, responsible of Cu^+^ mobilization to the secretory compartment. (*) The Ctr protein identified as the ScCtr2 ortholog is regulated in a MacA-independent manner and pumps copper ions to the cytosol from the vacuole compartment in copper scarcity conditions

The high-affinity copper uptake system in filamentous fungi contains all the elements mentioned above. In *Aspergillus fumigatus*, four Ctr proteins have been identified, AfCtrA1, AfCtrA2, AfCtrB, and AfCtrC. Phylogenetic analysis showed that these four proteins are closely related to *S*. *cerevisiae* Ctr1. AfCtrB showed higher homology to ScCtr2 (Park et al. 2014), a copper transporter that pumps copper ions out of the vacuoles in conditions of copper scarcity (Rees et al. 2004). Each protein has at least two transmembrane domains, four in the case of AfCtrB. All of them resemble the characteristic N-terminal Mets motifs for copper uptake and the MxxxM motif in one transmembrane domain, responsible of ion translocation (Park et al. 2014). In *A*. *nidulans*, three Ctr proteins have been identified, AnCtrA and AnCtrB and AnCtrC. AnCtrB is the putative ortholog of ScCtr2. The other two proteins possess the characteristic features of the copper transporting proteins. However, orthology studies of each Ctr protein reveal very different results. AnCtrC is related to many other Ctr proteins that have already been characterized, for example, ScCtr3 and the abovementioned AfCtrC. On the other hand, AnCtrA is related to no characterized protein so far (Antsotegi-Uskola et al., unpublished). Even though they have not been characterized, *A*. *nidulans* and *A*. *fumigatus* possess several putative plasma membrane reductases, responsible of iron and copper reduction prior to uptake. Two putative plasma membrane reductases are phylogenetically related to ScFre1 in *A*. *nidulans* and only one in *A*. *fumigatus*. In conclusion, both organisms possess all the components of the high-affinity copper uptake system described in *S*. *cerevisiae*: multiple Ctr proteins and the plasma membrane reductases (Antsotegi-Uskola et al., unpublished) (Fig. 1).

Out of all the Ctr proteins identified in each organism, usually, two function as copper uptake proteins. Although the contribution of each protein to the high-affinity copper uptake system varies, functional complementation between the activities is a general trait. In *A*. *fumigatus*, only *AfctrA2* and *AfctrC* were able to complement the disruption of *ctr1* in *S*. *cerevisiae* (Park et al. 2014). The same authors claim that both proteins are equally functional, since individual deletion mutants of *AfctrA2* and *AfctrC* exhibited a wild type–like phenotype with 100 μM BCS (Bathocuproinedisulfonic acid disodium salt, a copper chelator). According to Cai et al. (2017), however, the *AfctrC* deletion mutant showed defective colony morphology with 100 μM BCS, suggesting that this protein plays a dominant role in copper homeostasis.

Expression of Ctr proteins is copper-dependent, as expression of *AfctrA2* and *AfctrC* increases under copper deficiency conditions and progressively decreases as extracellular copper concentrations rise. According to Park and collaborators (Park et al. 2014), the expression of either *AfctrC* or *AfctrA2* is upregulated when the other is missing. Cai and collaborators (Cai et al. 2017), on the other hand, claim that only *AfctrC* expression is upregulated when the counterpart is deleted. Taken together, the data obtained by Cai and collaborators suggest that AfCtrC plays a dominant role in copper uptake. In both cases, the double deletion mutant displayed severe growth defects with 100 μM BCS, suggesting that AfCtrA2 and AfCtrC, the two Ctr proteins that work as copper internalization proteins, function in a complementary manner.

In *A*. *nidulans*, the *AnctrC* deletion mutant displayed a defect in pigmentation with 100 μM BCS (copper is required for the formation of conidiospores and colony pigmentation). On the other hand, *AnctrA* deletion had no visible effect on colony phenotype. The double deletion mutant showed a very aggravated growth defect in 100 μM BCS, suggesting a synergistic relationship between *AnctrA* and *AnctrC*. The short-term response, from basal condition to 3 h of exposure to copper and BCS of these proteins, has been studied and they show a different expression profile. The *AnctrC* transcript, as well as the protein, was present at copper satiety conditions, and expression increases with addition of BCS. The expression pattern of *AnctrA*, on the other hand, was different; the *AnctrA* transcript was present at copper satiety conditions, but the protein was not present. Upon addition of BCS, *AnctrA* transcript levels rose dramatically and the protein was first detected after 2 h of exposure to the copper chelator. The expression of both copper transporters was upregulated when the other one was missing, supporting the complementary role observed in protein function. In the case of *AnctrC*, the upregulation amounted to almost 17-fold, while in the case of *AnctrA*, it reached 64-fold. Despite this increase, *AnctrA* expression alone could not overcome the copper deficiency phenotype generated with 100 μM BCS. Correspondingly, the *AnctrC* deletant strain showed a pigmentation defect under copper starvation conditions. All together, these data suggest a dominant role of AnCtrC in *A*. *nidulans* high-affinity copper uptake system (Antsotegi-Uskola et al., unpublished).

The high-affinity copper uptake systems of *A*. *fumigatus* and *A*. *nidulans* share indeed many similar features with *S*. *cerevisiae*. Usually, more than one Ctr proteins are found in each organism but only one or two of them have a direct role in copper internalization. In both organisms, one of the Ctr proteins has a dominant role over the other one, but the deletion of both Ctr proteins proves the complementary relationship between both proteins.

## Copper detoxification

Even if copper import is a strictly controlled process, there are circumstances in which intracellular copper levels surpass the toxicity threshold. In response to an excessive intracellular copper concentration, detoxification/sequestration mechanisms are activated in order to restore cellular copper balance. In contrast to the similarity in proteins and mechanisms described for copper uptake, two major mechanisms have been reported for copper detoxification processes, marking an important division between filamentous fungi, as detailed below.

Two major mechanisms for copper detoxification have been described in fungi. One of them relies on copper sequestration by metallothioneins (MTs) and exhaustively described in *S*. *cerevisiae*. It involves two copper-specific MTs, Cup1 and Crs5, and these two proteins are responsible of copper resistance (Culotta et al. 1994; Jensen et al. 1996). This mechanism has not been described in *Aspergillus* species, but for example in *C*. *albicans*, metallothioneins do contribute to copper detoxification (Weissman et al. 2000).

The second mechanism, detoxification, has been described in other filamentous fungi studied so far. It relies on P_IB_-type ATPases, ATP-dependent heavy metal translocators that are deeply conserved from archaea to mammals. P_IB_-type ATPases have peculiar terminal extensions (N- or C-terminal) which contain metal (copper) binding domains (MBDs), rich in Cys and sometimes His. P_IB_-ATPases possess 6 to 8 transmembrane domains (TMDs), metal binding sequences in their TMDs, regulatory MBDs, a structure involved in enzyme phosphorilation (P-domain), a nucleotide binding domain (N-domain), and an energy transduction domain (A-domain) (Palmgren and Nissen 2011).

These copper extrusion pumps represent the main detoxification mechanism in bacteria (Ladomersky and Petris 2015), and in eukaryotes, they are also involved in copper compartmentalization into the secretory network where copper is incorporated into different Cu-dependent proteins as a cofactor. In humans, two P_IB_-ATPases ATP7A (Menkes disease protein) and ATP7B (Wilson disease protein) are responsible of delivery of copper to the *trans*-Golgi compartment (TGN) (Lutsenko et al. 2007). In response to Cu toxicity, both transporters change their location from the TGN to the cell membrane to act as detoxification (export) pumps conferring copper resistance to the cell (Suzuki and Gitlin 1999). In the dimorphic fungus *C*. *albicans*, each task is fulfilled by a different P_IB_-ATPase; CaCcc2 is involved in copper compartmentalization into the TGN and CaCrp1p is responsible of copper detoxification at the plasma membrane (Riggle and Kumamoto 2000; Weissman et al. 2000). It has recently been described that some species of the *Aspergillus spp*. possess two CaCrp1p homologs involved in copper detoxification (Yang et al. 2018).

This divergence in the mechanism of metal detoxification by different fungal species may be the result of an adaptation to their respective ecological niches. *S*. *cerevisiae* has been isolated from a range of environments (Goddard and Greig 2015), many of which are specific, such as fruits and flowers. In contrast, *C*. *albicans*, like most filamentous fungi, is an all-rounder found in environments ranging from the digestive tracts of animals to soils, where competition and antagonism are common. In these environments, P_IB_-ATPase (CaCrp1p) mediated copper export confers superior tolerance with respect to MTs, despite the greater cost in energy required to produce it and operate it. MTs, on the other hand, may allow for the immobilization of greater amounts of copper, as a resource, in media which may be especially poor in copper, such as flower nectar. In the same medium and growing conditions, *S*. *cerevisiae* is able to grow in up to 2 mM of CuSO_4_, whereas *C*. *albicans* tolerates 20 mM CuSO_4_ (Weissman et al. 2000).

*C*. *albicans* has become a benchmark for recent studies in filamentous fungi, such as the Aspergilli. Copper detoxification in *A*. *nidulans* relies on a P_IB_-type ATPase termed CrpA (Antsotegi-Uskola et al. 2017). AnCrpA contains all the characteristic domains described in a P_IB_-type ATPase: 8 TMDs, an ion translocation motif (CPC) in the sixth TMD and cysteine-rich MBDs in the cytoplasmic N-terminal domain. Five N-terminal MBDs are present as tandem repeats, and two CxxxC motifs are followed by three GMxCxxC classic heavy metal–associated domains (HMA). Copper detoxification in *A*. *fumigatus* also depends on a P_IB_-type ATPase termed AfCrpA (Wiemann et al. 2017).

Deletion of c*rpA* resulted in acute susceptibility to copper in *A*. *nidulans* and *A*. *fumigatus*. Susceptibility towards other metals was also observed in the *A*. *nidulans crpA* deletant strain, albeit not comparably with Cu susceptibility. Expression analysis corroborated the copper detoxification role of AnCrpA. Exposure to copper had a dramatic effect on *AncrpA* expression, while other metals barely induced expression. The *AncrpA* deletant strain manifested reduced resistance to Cu compared with the wild type strain in solid medium (~ 10-fold). With 100 μM CuSO_4_, the colony exhibited morphological defects and at 150 μM CuSO_4_, an almost complete growth inhibition. At 100 μM CuSO_4_, the colony exhibited a typical wild type radial growth pattern, but with very slight cellular density. This cellular morphology was named the “copper phenotype.” The *AncrpA* deletant strain showed some sensitivity to Cd and Ag, but far lower than to Cu (Antsotegi-Uskola et al. 2017).

*AncrpA* expression analysis revealed that it is principally expressed in the presence of copper (100 μM CuSO_4_), showing strong transient induction shortly upon exposure. mRNA appears 15 min after copper addition and the protein, after 30 min. The expression maximum is reached at 60 min followed by a gradual repression over time. The pattern of AnCrpA expression with Ag and Cd toxicities is similar, albeit several times weaker. In the case of Cd, with 250 μM Cd(NO_3_)_2_, a very faint band was visible at 120′ after exposure. It has recently been reported that AfCrpA also contributes to Zn resistance in *A*. *fumigatus* (Cai et al. 2018). These results indicate that even if it might have some role in other heavy metal detoxification, copper is the principal ion transported by CrpA (Antsotegi-Uskola et al. 2017).

**Fig. 2:**
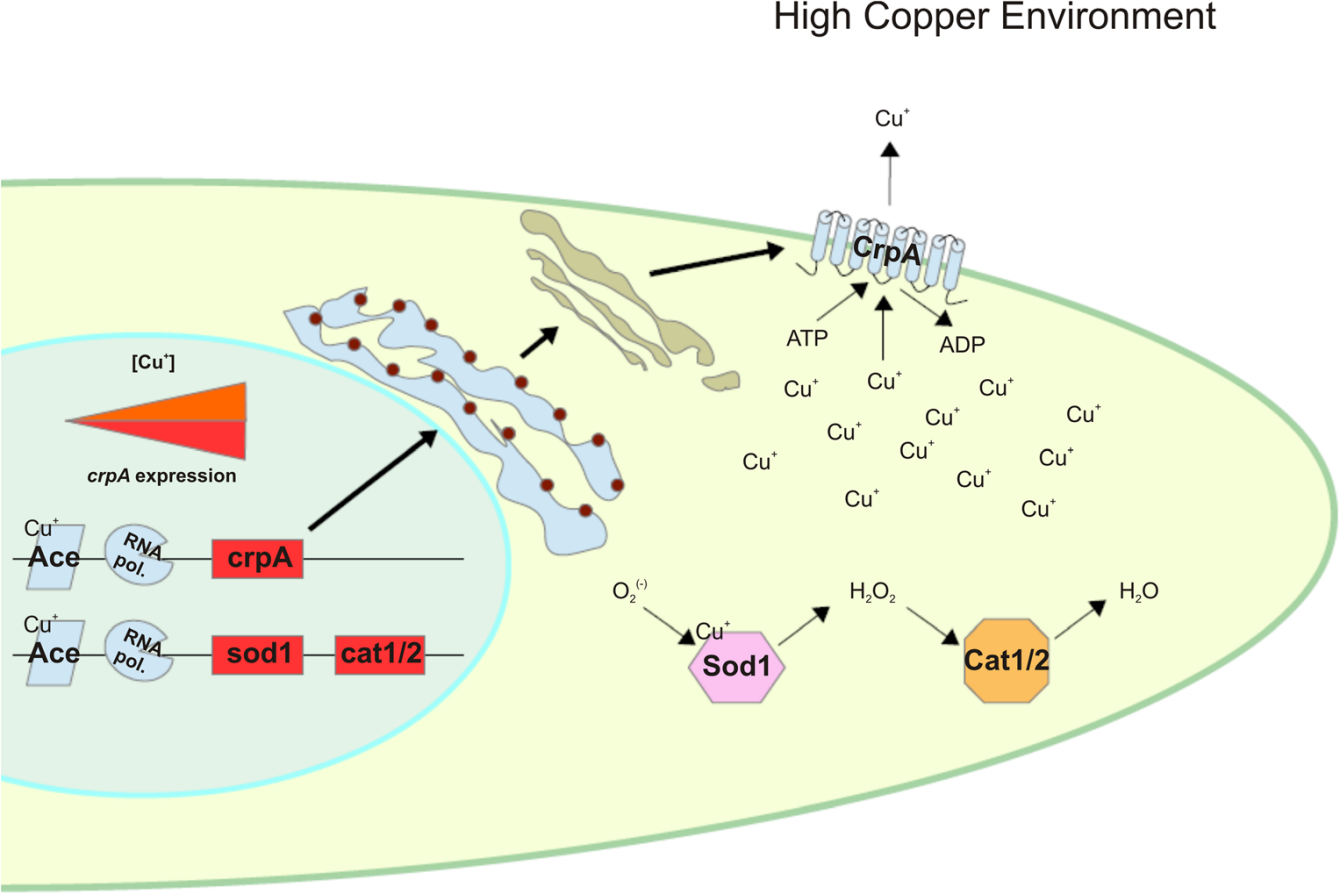
Copper homeostasis scheme for *A*. *nidulans* and *A*. *fumigatus*, under copper toxicity conditions. The TF AceA binds copper forming a tetra-copper-thiolate cluster, thereby changing its conformation. By changing conformation, AceA is able to bind DNA and enables the transcription of the P-type ATPase *crpA* coding gene. The P-type ATPase CrpA is first synthesized in the endoplasmic reticulum and migrates to the plasma membrane passing through the Golgi apparatus. Once stabilized in the plasma membrane, CrpA pumps Cu^+^ ions out of the cell in order to reduce copper toxicity within the cell. Meanwhile, the superoxide dismutase *sod1* and catalase *cat1/2* coding genes experiment AceA-mediated activation. Sod1 and Cat1/2 neutralize ROS generated by Cu^+^ toxicity or host organism defense mechanisms

AnCrpA is localized at the plasma membrane under copper stress conditions; however, depending on the level of stress, AnCrpA localization and function can change. After half an hour of copper addition to induce AnCrpA expression, subcellular localization studies show that fluorescence was localized at a reticulated network (highly likely the endoplasmic reticulum), surrounding structures that resemble the nucleus and strands associated to the plasma membrane under non-toxic copper loads. An hour after copper addition, AnCrpA appeared homogeneously dispersed in the cytoplasm and primarily distributed at the cell periphery, most likely at the plasma membrane. In numerous cells, fluorescence was polarized to the tip of hyphae. Approximately 2 h after copper addition, the protein mostly remained at the periphery, but fluorescent aggregates gradually appeared throughout the cytosol. After 3 h of copper exposure, large aggregates on vesicles resembling vacuolar compartments became visible (Antsotegi-Uskola et al. 2017). The authors hypothesized that de novo synthesized AnCrpA was translocated from the Golgi compartment to the plasma membrane upon copper toxicity. Once the detoxification process decreased intracellular copper levels, part of the AnCrpA localized to the PM may have been recycled by endocytosis and redistributed to the multivesicular body. It is also possible that AnCrpA could be recycled back to the PM (Pase et al. 2004). As a result of this, the fluorescent protein detected at the plasma membrane could represent a mixed pool of the de novo synthesized and recycled protein (Fig. 2).

The P-type ATPase termed CrpA exerts the main detoxification mechanism found in *A*. *nidulans* and *A*. *fumigatus*. Deletion of *AncrpA* resulted in hypersensitivity to Cu and a slight susceptibility to other metals. AnCrpA is mostly localized at the plasma membrane under copper toxicity conditions. However, AnCrpA localization varies throughout the detoxification process. Most filamentous fungi possess an ortholog of the above-described CrpA protein, meaning that their detoxification mechanisms could be P_IB_-ATPase-dependent.

## Genetic regulation of copper homeostasis

Copper homeostasis is principally regulated at the transcriptional level. Metal responsive transcription factors are able to sense the copper concentration within the cell and orchestrate a response by activating the copper import or copper detoxification, as required. These two regulatory paths are under the control of two separate transcription factors (Keller et al. 2005) which were first discovered in *S*. *cerevisiae* as Mac1 and Ace1 (Balamurugan and Schaffner 2006), and later described in *A*. *nidulans* and *A*. *fumigatus* (Antsotegi-Uskola et al. 2017; Cai et al. 2019; Kusuya et al. 2017; Wiemann et al. 2017).

The high-affinity copper uptake system is controlled by the transcription factor MacA. All ascomycetes possess the characteristic functional domains of this copper sensing TF. The N-terminal region contains a RGHR and GRP motifs and the “Cu fist” domain, all together, they are responsible of the Cu-dependent DNA binding of the TF (Cai et al. 2017). A C-terminal copper binding domain with two cysteine-rich motifs is responsible for copper sensing (Kusuya et al. 2017). Site-directed mutagenesis studies in *A*. *fumigatus* and *A*. *nidulans* have demonstrated that Cys residues of the N-terminal “Cu fist” domain are essential for MacA DNA binding (Cai et al. 2019). Under copper limitation, AfMacA binds copper response elements 5′-TGTGCTCA-3′ in the gene promoter regions and enables the transcription of the Ctr proteins for copper uptake (Park et al. 2017). On the other hand, copper accumulation leads to AfMacA inactivation. The C-terminal copper binding domain acts as an auto-inhibitory domain (Park et al. 2017). Different studies have demonstrated that AfMacA is necessary for Ctr protein expression, Cu^2+^ metalloreductase expression, SOD activity, and many other copper-dependent cellular processes (Fig. 1). It has recently been reported that AfMacA also regulates iron homeostasis and its localization in the cell varies depending on copper and iron concentration (Park et al. 2018). *AfmacA* disruption generated a copper starvation phenotype with manifest growth defects in copper depletion conditions (Cai et al. 2017; Kusuya et al. 2017; Park et al. 2017).

The copper detoxification process is orchestrated by the transcription factor AceA. In *A*. *fumigatus*, AfAceA is also involved in zinc detoxification (Cai et al. 2018). The characteristic domains that identify this TF are the “Cu fist” DNA binding domain, and the numerous cysteine residues arranged in CxC-CxxC segments through the protein sequence, identified as necessary for function in *S*. *cerevisiae* (Hu et al. 1990). Within the DNA binding domain, two different motifs are found, a 3 x Cys-His zinc finger and the KGRP motif for DNA binding stabilization. A single cysteine-rich domain is located downstream of the DNA binding domain. Under excess copper conditions, four Cu^+^ atoms bind the Cys-rich domain to form a tetra-copper-thiolate cluster (Dameron et al. 1991). Cluster formation leads to a conformational change that enables AceA-DNA binding. In this way, AceA enables the transcription of the copper detoxification machinery, principally *crpA*. AfAceA also regulates ROS detoxification genes *sod1*, *cat1*, and *cat2* (Wiemann et al. 2017), as well as the TFs *AftA* and *Yap1* mediating ROS defense (Hagiwara et al. 2016) (Fig. 2). *aceA* deletion resulted in a defective phenotype with severe growth defects under copper excess conditions in *A*. *fumigatus* and *A*. *nidulans* (Antsotegi-Uskola et al. 2017; Cai et al. 2018).

In summary, even though copper homeostasis is transcriptionally regulated by a single transcription factor in some organisms, such as *C*. *neoformans*, in most filamentous fungi, it is orchestrated by two transcription factors. The TF-termed MacA is responsible of conducting the copper internalization process and AceA does the same with copper detoxification. The characteristic features of these transcription factors are well conserved in filamentous fungi.

## Copper as a virulence factor

*A*. *fumigatus* is one of the most lethal airborne fungal pathogens responsible of severe invasive aspergillosis (IA), especially in immunocompromised individuals (Cai et al. 2017). Copper is a recognized virulence factor, as it is the cofactor of many enzymes that contribute to virulence, such as laccases or SOD. On the other hand, host organisms have developed defense strategies against fungal pathogens that target copper availability. By scavenging all Cu^+^ in the infection area, copper deprivation can be induced in the pathogen. On the contrary, host innate immune cells, such as macrophages, are able to mobilize copper to invading fungal tissue as a defense mechanism (Garcia-Santamarina and Thiele 2015). The generation of Reactive Oxidative Species (ROS) is another defense mechanism employed by the innate immune system. The superoxide dismutase (SOD) enzymes are copper-dependent enzymes responsible of neutralizing ROS, so the SOD enzyme is considered as an important virulence factor in organisms, like *C*. *albicans* and *C*. *neoformans* (Frohner et al. 2009; Narasipura et al. 2005). In conclusion, copper homeostasis plays a key role to overcome host barriers and the development of pathogenesis.

Disruption of any of the abovementioned TFs responsible for copper homeostasis has a direct effect on virulence. Recent studies revealed that AfMacA is necessary for normal virulence in *A*. *fumigatus* (Cai et al. 2017). *AfmacA* deletion results in delayed growth and pigmentation deficiency by DHN melanin synthesis inhibition. Laccases AfAbr1 and AfAbr2 are involved in melanin biosynthesis and they are copper-dependent proteins (Upadhyay et al. 2013). When copper uptake is impaired, laccase activity is substantially reduced (Park et al. 2014). Melanin confers a non-immunogenic status to the fungus. Inappropriately melanized conidia therefore become immunoreactive (Pihet et al. 2009). Melanin also provides a protective layer to the action of host-derived ROS (Jahn et al. 2000). *A*. *fumigatus* copper transporting protein expression is upregulated in the presence of human neutrophils (Sugui et al. 2008). This underscores the importance of copper uptake for pathogen viability within the host.

AfAceA has also been shown to be an important virulence factor. As detailed above, this TF controls the expression of the P_IB_-type ATPase *AfcrpA* responsible of copper detoxification in *A*. *fumigatus*. Moreover, AfAceA regulates the expression of catalases such as *Afcat1* and *Afcat2* involved in ROS neutralization and the TF *AfatfA* involved in ROS-response (Hagiwara et al. 2014). It has been reported that *AfatfA* deletion renders *A*. *fumigatus* avirulent (Pereira et al. 2017). *AfaceA* deletion represses *AfatfA* expression considerably, together with *Afcat1* and *Afcat2*, making the fungus more vulnerable to oxidative stress (Wiemann et al. 2017). Deletion of *AfaceA* inhibits the expression *AfcrpA*, the main copper detoxification system in *A*. *fumigatus*, leaving the fungus exposed to copper mobilization to fungal tissue by host innate immune system cells. Thus, the copper detoxification machinery is a key factor in pathogen viability during infection.

*Botrytis cinerea* is a necrotrophic fungal plant pathogen of worldwide distribution, capable of infecting a wide range of hosts. It is probably the best studied necrotrophic plant pathogen. Copper-dependent proteins play a central role in many aspects of the *B*. *cinerea*, including pathogenesis. The P-type ATPase BcCcc2, an ortholog of the *S*. *cerevisiae* Ccc2 copper transporting P-type ATPase that delivers Cu to the secretory compartment for subsequent protein modification (Smith et al. 2017), is crucial for virulence in *B*. *cinerea* (Saitoh et al. 2010). The *Bcccc2* deletion strain presented melanization and morphogenesis defects. Moreover, the deletion strain was not able to penetrate and infect host organisms (Saitoh et al. 2010). The most probable reason for this could be that the BcCcc2 targeted proteins could get no copper, and for this reason, all these cellular processes were impaired (Saitoh et al. 2010). *B*. *cinerea* is able to break through healthy tissue and secrete enzymes and toxins that generate necrosis (van Kan 2006). Lopez-Cruz and collaborators (Lopez-Cruz et al. 2017) revealed the importance of the Cu-Zn SOD in this process. The host oxidative burst (O^−^), a plant defense mechanism against pathogen organisms, plays an active role in *Botrytis* infection (Rossi et al. 2017). The O_2_^−^/H_2_O_2_ ratio in the necrotic lesion area is very significant for infection development. Higher concentration of H_2_O_2_ favors infection. BcSod1 actively generates H_2_O_2_, which damages plant tissue and reduces plant defense. The absence BcSod1 reduces the capacity to generate necrotic lesions in plant tissues as the oxidative environment is not appropriate for *Botrytis* infection development. The measured O^−^/H_2_O_2_ ratio in necrotic lesions caused by a *ΔBcsod1* strain is too high for infection. Incorrect function of BcCcc2 results in a defective BcSod1 function, as well as other proteins, proving the importance of the copper homeostasis system for *B*. *cinerea* virulence.

In summary, copper homeostasis plays an essential role in *A*. *fumigatus* virulence development. The high-affinity copper uptake system and the copper detoxification system are key factors in the infection mechanism. The high-affinity copper uptake system enables the maturation of many copper-dependent enzymes for virulence. On the other hand, the detoxification system confers the organism the necessary resistance for survival. In the necrotrophic fungus *B*. *cinerea*, the copper transporting P-type ATPase BcCcc2 is necessary for successful virulence development. Hence, copper homeostasis appears to be required for the development of infection in every major pathogenic fungus described thus far.

## Conclusions

The study of copper homeostasis in filamentous fungi has unveiled the relevance of this cation in growth, development, and pathogenicity. In the last years, there have been some contributions in the pathogenic fungus *A*. *fumigatus* and the model organism *A*. *nidulans*, but many details remain to be uncovered. Considering the repercussion of copper homeostasis on fungal biology, it is a topic worth studying in more depth, and in a wider range of species. The copper uptake and detoxification mechanisms and their respective transcription factors have been the focus of recent publications, identifying the principal participants of each process, but the copper buffering and intracellular distribution mechanisms remain practically unknown. Phylogenetic analyses have revealed the presence of proteins that act as copper scavenging and trafficking vehicles in other organisms (Ding et al. 2014), but so far, those proteins have not been investigated. Up to date, there is no evidence of CrpA homolog–dependent virulence in a phytopathogenic fungus. However, the BcCcc2 P-type ATPase, responsible of Cu traffic to the secretory compartment, in *Botrytis cinerea* has been proved to be essential for virulence. This evidence calls for further investigation covering intracellular copper trafficking and storage in oncoming years.

Copper has long been used as an antimicrobial agent in many different areas (Judet-Correia et al. 2011); however, it has often been applied in doses which cause severe impact on the environment. This excessive use has also brought about increasing levels of copper resistance in microbial pathogens. The mechanisms involved in fungal copper resistance are now in need of a thorough assessment in order to find new and effective methods to complement or replace copper in the control of fungal disease. New details on the mechanism of copper toxicity on fungal cells may shed light on the potential use of synergists affecting copper homeostasis that could help lower the currently required copper dose. The acquisition of copper by fungi in the soil, and its exchange between microorganisms and plants in symbiotic relationship, is another aspect which should be considered, to ensure sustainable agricultural and environmental conservation programs.
